# The Effects of Short-Lasting Anti-Saccade Training in Homonymous Hemianopia with and without Saccadic Adaptation

**DOI:** 10.3389/fnbeh.2015.00332

**Published:** 2016-01-05

**Authors:** Delphine Lévy-Bencheton, Denis Pélisson, Myriam Prost, Sophie Jacquin-Courtois, Roméo Salemme, Laure Pisella, Caroline Tilikete

**Affiliations:** ^1^Neuroscience Research Center – Institut National de la Santé et de la Recherche Médicale U 1028 – Centre National de la Recherche Scientifique UMR 5292Bron, France; ^2^Unité de Neuro-ophtalmologie, Hospices Civils de Lyon, Hôpital Neurologique Pierre WertheimerBron, France; ^3^University Lyon 1Lyon, France; ^4^Hospices Civils de Lyon, Hôpital Henry GabrielleSaint Genis-Laval, France

**Keywords:** compensatory training, lateral homonymous hemianopia, reading, saccadic adaptation, visual exploration

## Abstract

Homonymous Visual Field Defects (HVFD) are common following stroke and can be highly debilitating for visual perception and higher level cognitive functions such as exploring visual scene or reading a text. Rehabilitation using oculomotor compensatory methods with automatic training over a short duration (~15 days) have been shown as efficient as longer voluntary training methods (>1 month). Here, we propose to evaluate and compare the effect of an original HVFD rehabilitation method based on a single 15 min voluntary anti-saccades task (AS) toward the blind hemifield, with automatic sensorimotor adaptation to increase AS amplitude. In order to distinguish between adaptation and training effect, 14 left- or right-HVFD patients were exposed, 1 month apart, to three trainings, two isolated AS task (Delayed-shift and No-shift paradigm), and one combined with AS adaptation (Adaptation paradigm). A quality of life questionnaire (NEI-VFQ 25) and functional measurements (reading speed, visual exploration time in pop-out and serial tasks) as well as oculomotor measurements were assessed before and after each training. We could not demonstrate significant adaptation at the group level, but we identified a group of nine adapted patients. While AS training itself proved to demonstrate significant functional improvements in the overall patient group, we could also demonstrate in the sub-group of adapted patients and specifically following the adaptation training, an increase of saccade amplitude during the reading task (left-HVFD patients) and the Serial exploration task, and improvement of the visual quality of life. We conclude that short-lasting AS training combined with adaptation could be implemented in rehabilitation methods of cognitive dysfunctions following HVFD. Indeed, both voluntary and automatic processes have shown interesting effects on the control of visually guided saccades in different cognitive tasks.

## Introduction

Homonymous Visual Field Defects (HVFDs) refers to binocular deficits of lateral visual field, involving either half field (Homonymous Hemianopia) or quarter field (Homonymous Quandranopia). If the deficit persists after 6–8 months following a stroke, it is considered as a chronic visual disorder (Hier et al., 1983; Zhang et al., 2006). Such patients keep having difficulties in high-level cognitive functions such as reading (Zihl, 1995a) or exploring a visual scene (Zihl, 1995b). Disorganized patterns of eye movements might also contribute to the functional problems (Kerkhoff et al., 1992). Compensatory eye-movement strategies can spontaneously take place with time (Zangemeister et al., 1995; Pambakian et al., 2000) but rarely allow patients to reach healthy subjects' performance (Machner et al., 2009). Therefore, during the last decades many rehabilitation techniques have been developed. Among them, the compensatory method, aiming at facilitating large saccades into the blind hemifield in order to bring targets in the normal hemifield, is mainly recommended by experts (Bouwmeester et al., 2007).

Top-down strategy, based on explicit instructions and voluntary saccade training, has demonstrated functional improvements accompanied by oculomotor changes in the visual exploration (Kerkhoff et al., 1994; Zihl, 1995b) or reading (Zihl, 1995a) tasks. However, improvements are usually restricted to the trained ability and do not transfer to other tasks (Schuett et al., 2012). Furthermore, this strategy, where cognitive control is required to improve performance, requires repeated training sessions over months.

Bottom-up strategy relies on implicit oculo-motor training via sensory stimulation. Using a combination of auditory and visual stimuli (Passamonti et al., 2009; Keller and Lefin-Rank, 2010) or creating an optokinetic nystagmus thanks to presentation of a right-to-left moving text (Spitzyna et al., 2007), this strategy has already demonstrated promising results, with a transfer to both reading and visual exploration tasks, with a lower number of training sessions. A protocol combining visual pursuit and target jump toward the blind field has even shown to enhance functional performance following a single 30 min training (Jacquin-Courtois et al., 2013). Therefore, bottom-up strategy represents potentially more efficient and less costly rehabilitation of HVFDs.

Saccadic adaptation has been used for decades as a tool to explore plasticity mechanisms in animal models and humans (see Hopp and Fuchs, 2004; Pélisson et al., 2010 for reviews). It can be induced when subjects perform a series of saccade toward a visual target, which is shifted during the movement, producing a systematic post-saccadic error, which simulates the visual consequence of inaccurate saccades (McLaughlin, 1967). When the target is shifted away simulating short saccades, automatic corrective saccades are elicited. The repetition of such post-saccadic error signals over hundreds of trials is enough to implicitly trigger plasticity mechanisms increasing the amplitudes of saccades. This saccadic adaptation procedure could therefore represent an efficient bottom-up rehabilitation method in order to increase the amplitude of saccades made toward the blind field in HVFD patients. However, since in HVFD patients the target cannot be presented in the blind hemifield, we choose to apply the above procedure to an anti-saccade (AS) task in which subjects have to perform a saccade toward the direction opposite (blind hemifield) to the hemifield where the visual target is presented (healthy hemifield), but with the same amplitude (Hallett, 1978). We recently described in normal subjects that a version of this task with an outward target shift (more eccentrically) occurring at the completion of the AS is capable of adaptively increasing the amplitude of anti-saccades (Lévy-Bencheton et al., 2013).

The objective of this study was to test the effects of this short-lasting saccadic training (15 min), in which we take advantage of the oculo-motor plasticity mechanisms of visuo-motor adaptation in the context of voluntary AS training. In order to distinguish between the effects of the AS training (top–down method since AS involve the inhibition of the automatic saccade toward the peripheral visual target) and of the visuo-motor adaptation elicited by specific feedback target presentation (bottom-up method), 14 left- and right-HVFD patients were randomly submitted to three different 15 min AS tasks separated by 4 or 5 weeks, only one being designed to trigger outward oculo-motor adaptation of saccades made toward the blind field. Immediate re-appearance of the saccade target systematically shifted further away with respect to eye landing position at the saccade offset is known to trigger implicitly plasticity mechanisms increasing saccade amplitude (Adaptation), contrary to delayed or un-shifted re-appearance of the saccade target (Fujita et al., 2002; Lévy-Bencheton et al., 2013) which correspond to control conditions to test for the specificity of the effects of bottom-up adaptation mechanisms.

Visual Exploration (Pop-out and Serial) and Reading tasks were performed immediately before and after each AS task, as well as a Visual Function Questionnaire, to evaluate the functional and oculomotor effects of the three different trainings.

## Materials and methods

### Participants

Seventeen patients with a chronic HVFD after a stroke were asked to attend the inclusion visit (V0). Each patient underwent neurological and ophthalmological clinical examinations and assessment of the 30° central visual field (automated static system, Metrovision®, Pérenchies, France). A neuropsychological assessment of unilateral spatial neglect was performed during V0, including 10 trials of 20 cm line bisection test (Harvey and Milner, 1995), a stars cancelation test from the Behavioral Inattention Test (Halligan et al., 1991), a spontaneous daisy drawing and a clock test. None of them were under medications altering cognitive functions required for the task. All patients signed the written informed consent to participate to the study. Approval of all procedures was received from the National French ethical committee on human experimentation (Agence Nationale de Sécurité du Médicament et des produits de santé (ANSM) and Comité de Protection des Personnes (CPP) Sud-Est III), in agreement with French law (March 4, 2002) and the Declaration of Helsinki (n° 2008-057B).

**Figure 1:**
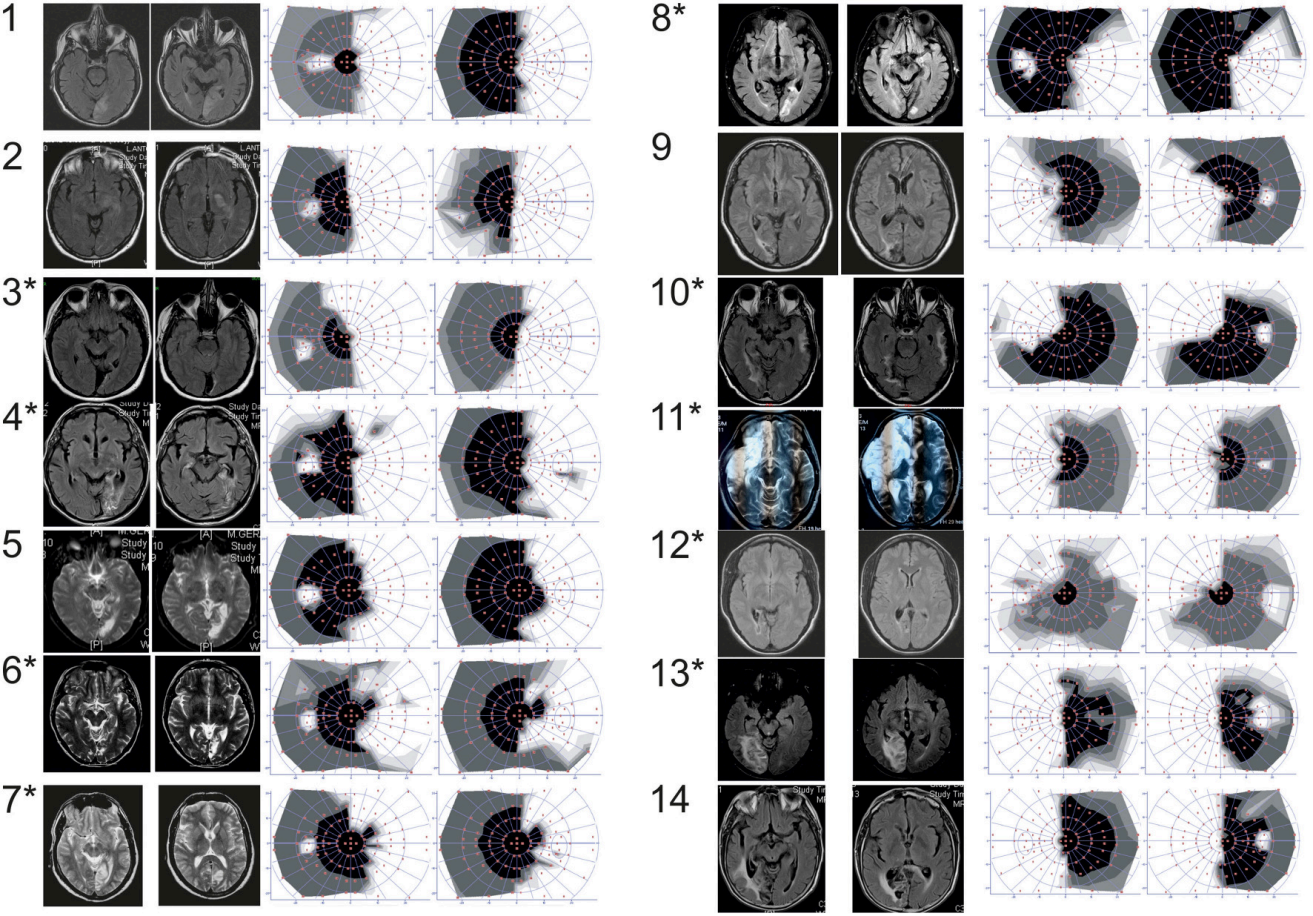
**Patients number**. Patients with (^*^) or without (no ^*^) positive slope, MRI (either T2 or FLAIR axial sequences) and 30° central visual field (automated static system, Metrovision®, Pérenchies, France) in the overall group of patients. White part of the visual field refers to the blind part of the visual field.

According to inclusion and exclusion criteria (Table 1) checked during the inclusion visit, 14 patients participated to the study (mean age 57 ± 10.51 y.o.; range 38–78; nine men and five women). Eight patients presenting a right-HVFD, six a left-HVFD, were included at least 6 months following an ischemic stroke (Figure 1). Clinical data of the patients are summarized in Table 2.

**Table 1:** **Inclusion and exclusion criteria assessed at the inclusion visit (V0)**.

**Inclusion criteria**	**Exclusion criteria**
✓ Patient with right- or left-HVFD ✓ Age: 18–80 years (included) ✓ Etiology: ischemic stroke ✓ Post stroke delay: at least 6 months ✓ Single lesion demonstrated on MRI ✓ Far and near visual acuity ≥5/10 ✓ Understanding the experimental recommendations ✓ Sitting position possible for 2 h ✓ Patient consenting to the study	✓ Visuo-spatial neglect ✓ Ophthalmologic (monocular visual acuity ≤ 4/10; strabismus, diplopia; ocular instability; nystagmus; maculopathy; glaucoma; retinopathy; ongoing orthoptic rehabilitation) ✓ Neurologic (understanding disorder; degenerative neurologic disorder; epilepsia; severe handicap not allowing sitting position for 2 h or concentrate for 30 min) ✓ Not French reader ✓ Non-stabilized medical affection ✓ Pregnancy ✓ Patient unable to sign consent

**Table 2:** **Clinical data of the patients**.

**Patients**	**Sex**	**Age**	**Delay in months**	**Cerebral artery territory**
1	M	67	30	Posterior
2	M	57	48	Middle
3	F	44	78	Posterior
4	M	66	12	Posterior
5	M	63	84	Posterior
6	F	52	180	Posterior
7	M	59	120	Posterior
8	M	78	7	Posterior
9	M	53	36	Posterior
10	M	60	138	Posterior
11	F	50	108	Middle
12	F	38	96	Posterior
13	F	50	72	Posterior
14	M	67	12	Posterior

Patients 1–8 represent right-HVFD and patients 9–14 represent left-HVFD following a left and right hemisphere lesion, respectively.

### Study design

**Figure 2:**
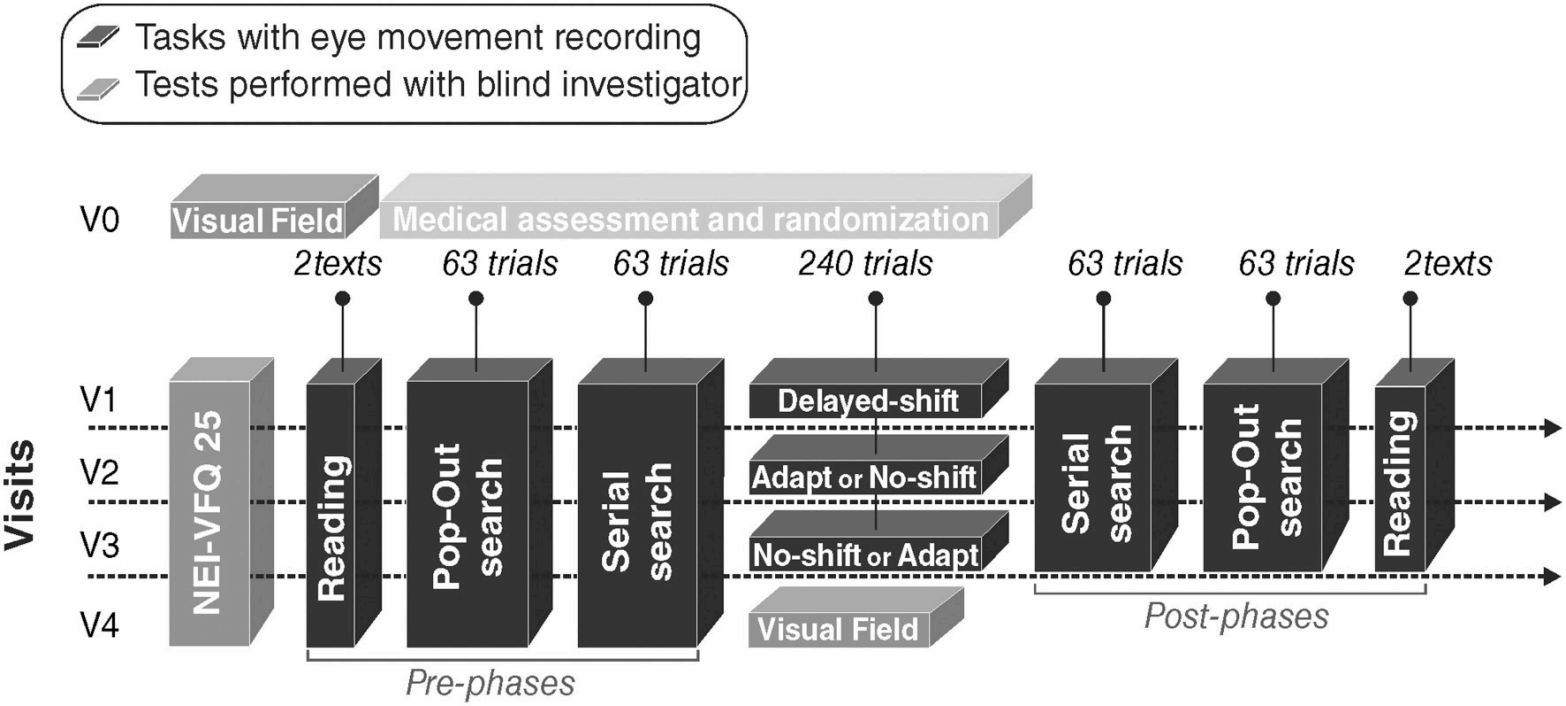
**Study design**. Each patient came first for an inclusion visit (V0) and then four times: three visits with a training (V1, V2, and V3) plus a follow-up visit (V4). V1 occurred 1–6 weeks after V0 and an interval of 4 or 5 weeks spanned between each of the four following visits. Black blocks correspond to the eye movements recording tasks. Gray blocks correspond to tests performed with a blind investigator. Light gray block corresponds to the medical assessment and the randomization of training.

After the inclusion visit V0, the patient came for three successive visits (V1, V2, and V3) each separated by 4–5 weeks. During each visit, one out of the three computerized-training (Delayed-shift, Adaptation, No-shift) was tested. Delayed-Shift training was systematically performed in V1, so that each patient could learn how to proceed with the training task (more details below). During V0, within each group of patient with right- or left-HVFD and with or without macular sparing, the patients were randomly attributed the training order for V2 and V3 (Adaptation or No-Shift training) (Figure 2). A fourth follow-up visit (V4) was organized after V3. The whole duration of the protocol for a given patient was between 14 and 21 weeks. Each visit started with the NEI-VFQ 25 (National Eye Institute 25-Item Visual Function Questionnaire, 2000) systematically assessed by a blind investigator. The three training visits (V1, V2, and V3) included the training period and immediate pre- and post-visual exploration and reading phases. A last assessment of NEI-VFQ 25, visual exploration and reading performance, and visual field was performed during the last visit (V4).

### Eye movement recording and analysis

Horizontal and vertical eye positions were continuously recorded during visual exploration, reading and training, using an infrared Eye-Tracker system (Cambridge Research System, Cambridge, UK). The infrared camera was mounted above a chin-rest and allowed high frequency (250 Hz) acquisition of the eye images which were reflected by a 45° tilted mirror located in front of the subject. The patient was seated in front of a computer screen (140 Hz vertical refresh rate), his head maintained by the chin-rest at 57 cm from the screen. Patient's eye position was calibrated using a nine-point grid at the beginning of each session (or anytime he/she moved the head).

All horizontal and vertical saccades were analyzed offline using a laboratory-developed program under Matlab version 7.8 (Mathworks, MA, USA) and this automatic analyze was manually checked and corrected by the experimenter if needed.

### Pre- and post-phases

#### Visual exploration tasks

**Figure 3:**
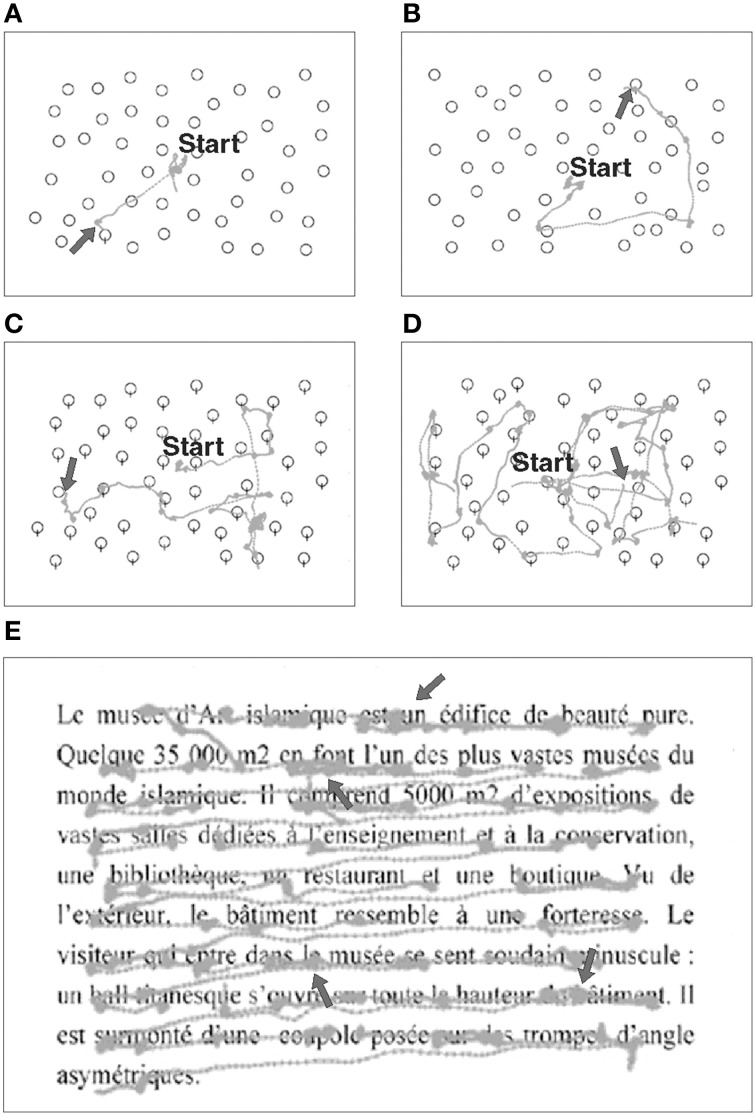
**Examples of eye movement recordings during visual exploration and reading tasks in a patient (#8) with right-HVFD**. Gray traces represent raw eye movements performed by the patient. In the Pop-out **(A,B)** and Serial **(C,D)** exploration tasks, arrow signals the endpoint of the saccade made to the target. When the target is in the healthy hemifield (**A,C**) fewer exploration saccades are made as compared to when the target is in the blind hemifield (**B,D**). In the reading task **(E)**, arrows point to the typical oculomotor deficits of right HVFD patients, i.e., smaller saccades with higher rate of fixations.

For both *Pop-out and Serial exploration* tasks, 63 images (20° horizontal, 15° vertical) representing black-edges balloons on a white background were successively presented on the screen, with a variable amount of stimuli (12, 24, or 48). Patient was asked to find a target among distracters, and pushed a button as soon as he found the target or another button if no target was found. The target was present in 20 trials and absent in one trial per stimulus difficulty (amount of distracters). In the Pop-out exploration task, the patient had to find a balloon with a string among balloons without strings (Figures 3A,B). In the Serial exploration task, he had to find the only balloon without string among balloons with strings (Figures 3C,D; Morris et al., 2004).

#### Reading task

Two texts were successively presented to the patient. Each text was extracted from French newspaper, written in black letters on a white background, in Times New Roman font, size 30, justified (Figure 3E). Sets of three letters spanned 2° of visual angle. Each text had similar number of words (85–90 words per text), and had the same neutrality in order to avoid emotional bias. The patient was asked to silently read each text at his own speed, and had to push a button as soon as he finished reading. After reading the two texts, comprehension was confirmed by asking the patients to verbally summarize their content. A new text was presented at each session and visit in order to avoid any learning effect.

#### NEI-VFQ 25

The 25-item National Eye Institute Visual Functioning Questionnaire (NEI-VFQ 25) attests the quality of life (QOL) of patients with 25 different items focusing on vision and grouped in 12 subscales including one single-item subscale focusing on general health. Each subscale is scored within a 100-point scale with 100 indicating no difficulty and 0 indicating the worst difficulties. A composite score—mean score of all subscales except the general health item—is used.

### Training

**Figure 4:**
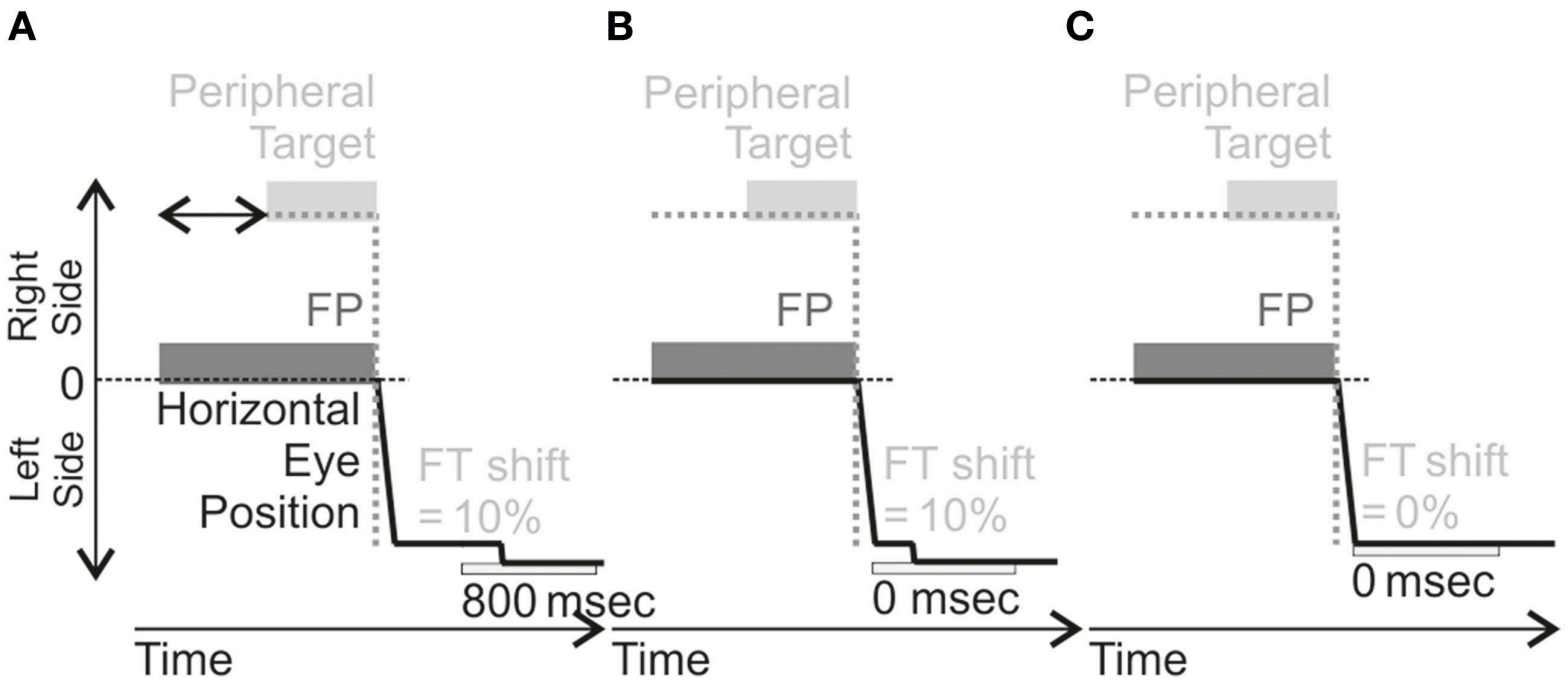
**Schema of AS training in the different training**. These illustrations are for Left-HVFD patients (leftward AS elicited by a target in the right visual field). For each training, the Fixation Point (FP) is first presented, followed by a peripheral target appearing between 1100 and 1500 ms (horizontal black arrow represented in A) after FP presentation, located on the right. In the Delayed-Shift training (**A**), the feedback target (FT) is presented 800 ms after AS completion at a location shifted outward by 10% of the anti-saccade amplitude. In the Adaptation training (**B**), the FT is also presented at the shifted location (same 10% offset) but at the time of anti-saccade completion (0 ms). In the No-Shift training (**C**), the FT is presented without delay from anti-saccade completion (0 ms) but also without offset, i.e., at the mirror position of the peripheral target. For Right-HVFD patients, the training was similar except that the peripheral target was now presented on the left side and anti-saccades performed to the right.

The training consisted of three different anti-saccades (AS) training: one with (Adaptation) and two without (Delayed-shift and No-shift) saccadic adaptation (Figure 4). More details of the general procedure and on these three trainings can be found in a previous study (Lévy-Bencheton et al., 2013).

#### Task common to the three trainings

Each patient gazed at a central red cross used as a Fixation Point (FP). After a random time (range: 1100–1500 ms), a peripheral target was presented in the healthy hemifield, on the horizontal meridian, randomly at 6°, 9°, or 12° lateral to the FP (overlap paradigm). The patient was instructed to execute an AS in the direction opposite to the target (thus in the blind hemifield), with equivalent amplitude. He had to react within a delay of 1400 ms after peripheral target presentation, which remains on the screen until detection of the saccade. The timing could be adapted to the patient's ability, usually by increasing the delay if the patient was too slow. Thanks to a gaze-contingent paradigm, the appearance and disappearance of stimuli on the screen were strictly controlled: as soon as the AS was detected, FP and peripheral target were extinguished by the software. Upon completion of the AS a “feedback” target (FT) was presented either at the mirror position of the peripheral target (No-Shift) or shifted 10% outward with respect to the executed saccade (i.e., shifted in the blind hemifield; Adaptation and Delayed-shift) depending on the actual training (see following paragraphs). To re-inforce saccade accuracy, a spatio-temporal criteria had to be met in order to present the feedback target: the saccade has to reach at least 90% of the mirror peripheral target distance (spatial threshold) within 600 ms (temporal threshold) after the initiation of the saccade following FP and target disappearance (more details in Lévy-Bencheton et al., 2013). If succeeded, a short and high-pitched “success” sound was presented to the subject. If the spatio-temporal criteria were not fulfilled, a longer low-pitched “error” sound occurred and the feedback target was not presented. After completion of the AS, patient was instructed to shift his gaze back to the center of the screen in preparation to the next trial. A total of 240 trials (80 for each target position) was presented. In case of excessive failure trials (above 20%), the total amount of presented trials was increased up to 300. Each training lasted around 15–20 min.

#### Differences between training

Only parameters concerning the feedback target were varied between the three trainings (Figure 4). For the adaptation training, the feedback target was systematically presented with an offset, with respect to final eye position, in the direction of increasing eccentricity (outward) and with an amount equal to 10% of the actual size of eye displacement (Figure 4B). This feedback target was turned on at the end of the anti-saccade without any delay (0 ms). For the delayed-shift training, the feedback target was presented at the offset location but delayed from the end of the anti-saccade by 800 ms (Figure 4A). For the No-shift training, the feedback target was presented without any delay but at a location, which corresponded to the mirror target position (Figure 4C).

### Data analysis

Computerized-tests (Visual exploration, reading) were submitted to two different kind of measurements. The *functional and oculomotor measures* represent parameters defining the subject's performance in the different tasks excluding eye movements (i.e., time needed to perform the visual exploration and reading tasks, measuring the Reaction time, word per minute, respectively) and parameters defining the subject's performance in the different tasks including eye movements (mean saccadic amplitude, mean fixation duration), respectively. The questionnaire of Quality of Life (NEI-VFQ 25) and the Visual Field were analyzed separately. We present below the main analysis, which specifically tested our hypotheses, and the complementary analyses, which were performed as follow-up when the main analysis provided significant results.

#### Main data and statistical analyses

##### Regression slope

The regression slope of the relationship, during all training, between primary saccade gain and trial number was measured for each patient separately, for each target eccentricity (6°, 9°, and 12°) and was then averaged across target eccentricities. To evaluate whether the saccadic gain evolved significantly over the time of training, we performed a one-sample *t*-test comparison of the averaged regression slopes to the standard value 0.

##### Predictive independent factors

Individual positive or negative slope during the adaptation training was used as a two levels predictive independent factor in all following functional and oculomotor statistical analysis, to test for the potential effect of gain change during the adaptation training (i.e., plasticity mechanisms). HVFD-side and Macular Spare are two factors, which can influence reading performance (Zihl, 1995a; Trauzettel-Klosinski and Brendler, 1998; Upton et al., 2003). Thus, these last two factors have been added as predictive independent factors in the ANOVAs related to reading.

##### Visual field

The mean macular threshold and the mean corrected deficit for the two eyes were calculated in each patient, separately for the inclusion visit (V0) and the last visit (V4). Paired *t*-test was performed to compare the *macular threshold and the mean corrected deficit*, separately, between the first (pre-phase: V0) and last visit (post-phase: V4).

##### Visual exploration tasks (Pop-out and Serial)

*Functional measures*. Reaction time (RT, in ms) was calculated for trials with target present as the period elapsing between the presentation of the image on the screen and the response of the patient (press button). A Two-way repeated measures ANOVA was performed on the RT, separately for the Pop-out and Serial exploration tasks, with the following dependent factors: Training (Delayed-Shift/Adaptation/No-shift) and Phase (Pre-phase/Post-phase) with the factor slopes (Positive/Negative) as predictive independent factor.

*Oculomotor measures*. Mean horizontal amplitude of saccades performed toward the treated-side and non-treated side were calculated over the entire block and averaged for each participant. Mean fixation duration before the forthcoming saccades toward the treated vs. non-treated side (beginning of the forthcoming saccade—ending of the previous saccade) were also calculated for each block and averaged for each patient. Mean horizontal saccadic amplitude and mean fixation duration were submitted to a Three-way repeated measures ANOVA, separately for the Pop-out and Serial exploration tasks, with the following factors: Saccade Direction (Treated/Non-treated side), Training (Delayed-Shift / Adaptation / No-Shift) and Phase (Pre-phase / Post-phase), with the factor slopes (Positive / Negative) as predictive independent factor.

##### Reading task

*Functional measures*. Reading time was calculated as the time elapsing between the eye fixating the very first word and the very last word of each text. Reading speed (word per minute) was calculated as the number of words read divided by reading time. A Two-way repeated measures ANOVA was performed on the word per minute (WPM) with the Training and the Phase as dependent factors, with the factor slopes (Positive/Negative) HVFD side (Left-HVFD/Right-HVFD) and macular spare (< 5°/>5°) as predictive independent factors.

*Oculomotor measures*. Mean horizontal amplitude of saccades performed toward the treated-side and non-treated side were calculated over the entire text and averaged for each participant. A Three-way repeated measures ANOVA was performed on the mean horizontal saccadic amplitude testing the effect of Training (Delayed-Shift/Adaptation/No-Shift), Saccade Direction (Treated side/Non-treated side) and Phase (Pre-phase/Post-phase). The slopes, HVFD side and macular spare were added as predictive independent factors. Note that here, Treated-side corresponds to leftward return saccades and Non-treated side to rightward reading saccades in left HVFD patients, whereas the opposite is true for right HVFD patients. Note also that for the return saccades, only those from the right-end of a line to the left-start of the next line were taken into account (Zihl, 1995a). Mean fixation duration and numbers of saccades before a forthcoming return saccade vs. a forthcoming reading saccades were also calculated for each text and averaged for each patient. Because of the different nature of the reading (rightward) vs. return (leftward) saccades (i.e., fewer return saccades as compared to the reading saccades, independently of the treated-side) these analysis were performed separately with the factors Training and Phase submitted to a Two-way repeated measures ANOVA excluding the treated-side factor.

##### NEI-VFQ 25

Composite scores measured during each visit were used to determine the potential effect of training on the patients' quality of life (QOL). Pre-phase scores were determined by the questionnaire at the beginning of the session while the corresponding post-phase scores were determined at the beginning of the next visit 1 month later. A One-way repeated measure ANOVA with four levels (i.e., pre-delayed/baseline, post-delayed, post-adapt and post-no-shift) was performed on the composite score with the dependent factors Training. Additionally, because of the different categories tested in that questionnaire (including reading component) we assessed whether the saccadic training influences the composite score of the NEIVFQ25 questionnaire. For these reasons, we thought relevant to add the slopes, the HVFD side and macular spare as predictive independent factors.

#### Complementary data and statistical analysis

In case of specific increase of performance immediately after the adaptation training, in functional *or* oculomotor parameters, we performed long-term analyses thanks to paired-*t* tests on the same parameter at 1 month. These long-term analyses compared the immediate post-phase following the adaptation training with the pre-phase at 1 month (visit V3 or V4).

Note that in case of specific *and* simultaneous (functional and oculomotor) increase of performance following the adaptation training, additional analyses were performed on oculomotor parameters such as number of saccades and were also checked at 1 month.

Statistical analyses were performed with the Statistica 10 software (Statsoft, Tulsa, OK). *Post-hoc* Least Significant Difference (LSD) Fischer was performed following a significant interaction in ANOVAs. Significant level was set at *p* < 0.05.

## Results

### Regression slope and adaptation marker

The slope of the relationship between gain of the anti-saccades and trial number during the three trainings did not differ significantly from zero at the group level, [*t*_(41)_ = −0.28, *p* = 0.78; *t*_(41)_ = 1.41, *p* = 0.16; *t*_(41)_ = −1.90, *p* = 0.06 for the Delayed-shift, Adaptation and No-shift training, respectively]. However, five patients had a negative (range from −0.001118 to −0.000080) and nine patients a positive slope (range from 0.000033 to 0.001294), five of whom had right-HVFD and four left-HVFD. The slope of the relationship between gain of the anti-saccades and trials numbers during the adaptation training differed significantly from zero at a group level, for patients who showed positive slope [*t*_(26)_ = 4.65, *p* < 0.01]. The signed slope of the relationship between gain and trial number during the adaptation training was used as a marker of the efficiency of the outward anti-saccade adaptation for the oculomotor statistical analyses (see Section Materials and Methods) and for the NEI-VFQ 25 questionnaire.

### Visual field

Mean macular threshold and mean corrected deficit did not differ at visit V4 (28.83 and 8.23 dB) as compared to the pre-phase (28.78 and 8.56 dB) [*t*_(13)_ = −0.07, *p* = 0.94 and *t*_(13)_ = −1.92, *p* = 0.07].

### Visual exploration tasks

#### Pop-out

##### Functional measures

**Figure 5:**
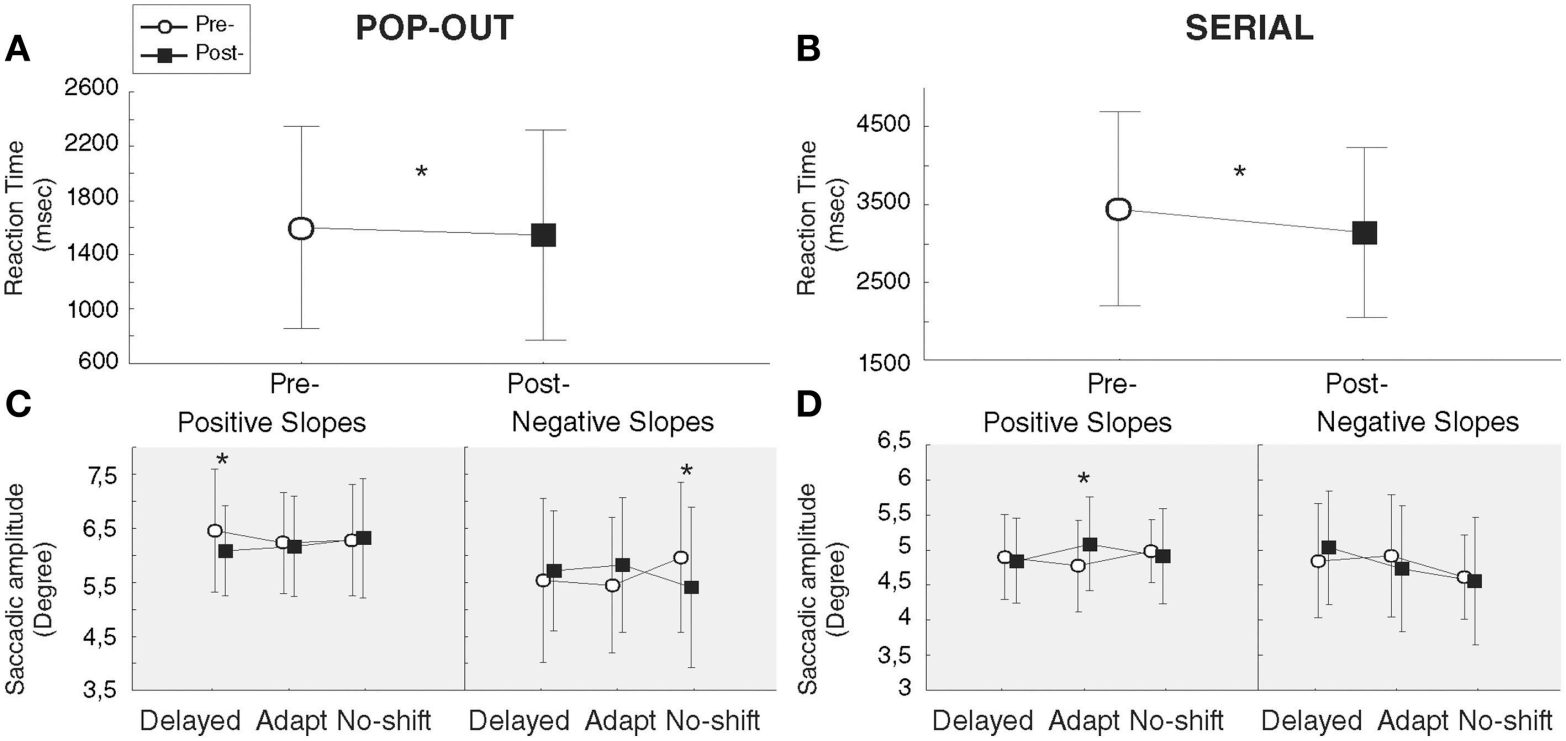
**Results of exploration tasks**. Functional **(A,B)** and oculomotor (**C,D**, gray background) measures of Pop-out **(A,C)** and Serial **(B,D)** exploration tasks. Bars represent the confident interval at 0.95. Stars represent significant differences between pre- and post-phases revealed by *Post-hoc* LSD Fisher test.

Following the Two-way repeated measures ANOVA with the factors Training and Phase, and the factor slopes as predictive independent factor, we observed a significant decrease of the RT following all training [main effect of Phase: *F*_(1, 12)_ = 5.17, *p* = 0.04]. Further, a significant Training × Phase interaction [*F*_(2, 24)_ = 3.78, *p* = 0.037] resulted from a significant decrease of the RT in post-phase [1504.6 ms] relative to the pre-phase [1650.4 ms] for the delayed-shift training only [*p* = 0.019]. Neither the decrease of RT in post-phase for the adaptation training [1473.9 pre-/1380.8 post-] nor the increase in the post-phase for the no-shift training [1436.4 pre-/1504.7 post] were significant (*p* = 0.12 and 0.25, respectively; Figure 5A).

##### Oculomotor measures

Despite a significant interaction between Training × Phase × Slope [*F*_(2, 24)_ = 4.76, *p* = 0.018], following the Three-way repeated measures ANOVA with the factors Training, Phase and Saccade Direction, and the factor slopes as predictive independent factor, there was no significant increase of saccade amplitude during the Pop-out exploration task in the group of patients who demonstrated a positive slope i.e., an increase of anti-saccade amplitude during the adaptation training [*p* = 0.70] (Figure 5C). The interaction was instead driven by a significant amplitude decrease of saccades following the delayed-shift training for this group [*p* = 0.04] and following the no-shift training for the group in which the adaptation training was inefficient (negative slope) [*p* = 0.02]. These changes were also supported by the three level interaction of the Saccade direction × Training × Phase [*F*_(2, 24)_ = 5.68, *p* = 0.009] showing that the significant amplitude decrease following the delayed-shift training occurs for the treated-side [*p* = 0.005] and for the non-treated-side following the no-shift training (*p* < 0.001). There was no change of fixation duration in either training (Phase effect [*F*_(1, 12)_ = 0.08, *p* = 0.77]; Training × Phase interaction [*F*_(2, 24)_ = 3.28, *p* = 0.055]; Table 3).

**Table 3:** **Mean fixation duration in ms (±SD) of the forthcoming saccades performed toward the treated- vs. non-treated side in visual exploration and reading tasks**.

	**Delayed-shift**		**Adaptation**		**No-shift**	
	**Pre-phase**	**Post-phase**	**Pre-phase**	**Post-phase**	**Pre-phase**	**Post-phase**
**POP-OUT**						
Treated-side	213.38	206.28	220.56	196.85	200.31	240.98
	(±38.09)	(±44.57)	(±88.93)	(±82.14)	(±44.64)	(±145.30)
Non-treated side	208.02	199.31	193.81	183.70	195.84	222.09
	(±41.59)	(±45.33)	(±49.57)	(±71.29)	(±45.81)	(±104.18)
**SERIAL**						
Treated-side	218.83	213.97	223.14	214.24	211.66	235.14
	(±41.70)	(±46.09)	(±76.34)	(±51.33)	(±48.64)	(±103.80)
Non-treated side	220.59	215.65	222.11	215.31	213.09	245.33
	(±53.12)	(±47.97)	(±62.85)	(±48.82)	(±48.90)	(±143.75)
**READING (BEFORE RETURN SACCADES)**						
Treated-side	206.76	232.88	230.93	228.93	225.40	233.28
(left-HVFD)	(±91.73)	(±102.05)	(±71.11)	(±66.94)	(±102.90)	(±130.42)
Non-treated side	294.53	230.48	271.86	266.61	259.50	275.56
(right-HVFD)	(±118.38)	(±44.98)	(±71.68)	(±86.02)	(±68.72)	(±87.28)
**READING (BEFORE READING SACCADES)**						
Treated-side	274.87	247.18	271.32	258.95	277.04	263.37
(right-HVFD)	(±61.86)	(±41.45)	(±66.21)	(±61.51)	(±66.23)	(±61.09)
Non-treated side	257.34	270.76	273.82	266.24	252.10	224.72
(left-HVFD)	(±68.87)	(±37.38)	(±82.70)	(±68.95)	(±71.79)	(±33.32)

#### Serial

##### Functional measures

Following the Two-way repeated measures ANOVA with the factors Training and Phase, and the factor slopes as predictive independent factor, we observed a main effect of Phase [*F*_(1, 12)_ = 20.15, *p* < 0.001], due to a significant decrease of RT in the post-phase [3148.2 ms] as compared to the pre-phase [3451.4 ms], independently of the training (no interaction; Figure 5B). In addition, we found a Training × Slope interaction [*F*_(2, 24)_ = 7.28, *p* = 0.003] in which the group of patients with positive slopes is faster than the group of patients with negative slopes to explore the visual scene in the adaptation [*p* = 0.04] and no-shift [*p* = 0.02] training, independently of the phase.

##### Oculomotor measures

Three-way repeated measures ANOVA with the factors Training, Phase and Saccade Direction, and the factor slopes as predictive independent factor has been performed and revealed a significant and specific increase of the saccadic amplitude following the adaptation training only, for the patients with positive slopes [*p* = 0.003] (Training × Phase × Slope interaction [*F*_(2, 24)_ = 5.69, *p* = 0.009]; Figure 5D). No significant interactions occur for the Saccade direction × Training × Phase [*F*_(2, 24)_ = 3.01, *p* = 0.068] nor the for the Saccade direction × Training × Phase × Slope [*F*_(2, 24)_ = 2.97, *p* = 0.07]. Concerning fixation duration, neither Phase effect nor Training effect nor Training × Phase interaction were demonstrated [*F*_(1, 12)_ = 1.16, *p* = 0.30; *F*_(2, 24)_ = 1.00, *p* = 0.38; *F*_(2, 24)_ = 2.53, *p* = 0.10, respectively] (Table 3).

### Reading

#### Functional measures

**Figure 6:**
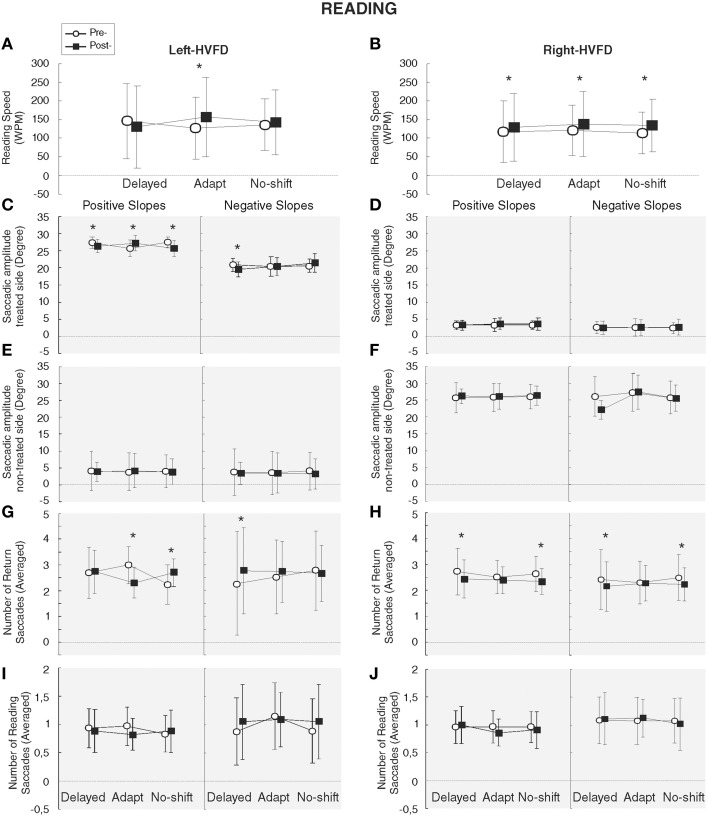
**Results of reading task**. Functional and oculomotor (gray background) measures are represented separately for the left-HVFD **(A, C, E, G, I)** and right-HVFD **(B, D, F, H, J)** patients. Oculomotor measures include the amplitude of saccades toward the treated-side (**C,D**), non-treated side **(E,F**) in degree and the number of return (**G,H**) and reading (**I,J**) saccades. Bars represent the confident interval at 0.95. Stars represent the significant difference between pre- and post-phases revealed by *Post-hoc* LSD Fisher test.

Two-way repeated measures ANOVA with the factors Training and Phase and the slopes, HVFD-side and Macular spare as predictive independent factors has been performed for the functional measures of the reading task. Analyses of the WPM could not be performed in one patient because we failed to identify the end of the reading due to a lack of eye-tracking signal (see Section Materials and Methods). Reading speed (WPM) of the 13 analyzed patients was significantly increased following the adaptation and the no-shift training (*p* = 0.0003 and 0.002, respectively), yielding a significant Training × Phase interaction [*F*_(2, 10)_ = 6.66, *p* = 0.014]. Additionally, the Training × Phase × HVFD-side interaction [*F*_(2, 10)_ = 4.50, *p* = 0.04] showed that in right-HVFD patients the reading speed increased following each training [125.6–139.5 WPM, *p* = 0.03; 130.5–148.6 WPM, *p* = 0.009; 121.5–142.5 WPM, *p* = 0.004 for Delayed-shift, Adaptation, and No-shift training, respectively], while in left-HVFD patients a specific increase of reading speed from 136.2 to 168 WPM was found after the adaptation training only [*p* = 0.001; *p* = 0.1; *p* = 0.1 for adaptation, delayed-shift, and no-shift training, respectively] (Figures 6A,B). Long-term analyses performed on the reading speed of left-HVFD patients at 1 month following the adaptation training visit did not reveal any difference with the initial post-adaptation measure [*t*_(4)_ = −2.13, *p* = 0.10], showing that the beneficial effects remained at long-term.

#### Oculomotor measures

Three-way repeated measures ANOVA performed on the oculomotor measures revealed a significant HVFD-side × Saccade direction interaction [*F*_(1, 5)_ = 404.61, *p* < 0.0001] led us to perform separate analyses for saccades in the treated-side and those in the non-treated side. Results for the treated-side saccades showed a significant increase of the amplitude of return leftward saccades for the left-HVFD patients with positive slopes following the adaptation training only [*p* = 0.001] [interaction Training × Phase × HVFD-side × Slope [*F*_(2, 10)_ = 7.68, *p* = 0.009] (Figure 6C). The absence of difference in the follow-up long-term analysis [*t*_(2)_ = −0.93, *p* = 0.45] suggests that this improvement is still present at 1 month. In addition, in the same patients (left-HVFD patients with positive slopes), we observed a decrease of the number of leftward saccades again specifically following the adaptation training (Training × Phase × HVFD-side × Slope interaction [*F*_(2, 18)_ = 6.26, *p* = 0.008]; Figure 6G) and again remaining stable at 1 month [t_(2)_ = 1.15, *p* = 0.37]. Mean fixation duration was unchanged both for the return saccades (Main effects, all *p* > 0.12; interactions, all *p* > 0.14) and the reading saccades (Main effects, all *p* > 0.53; interactions, all *p* > 0.08; Table 3).

### NEIVFQ 25

**Figure 7:**
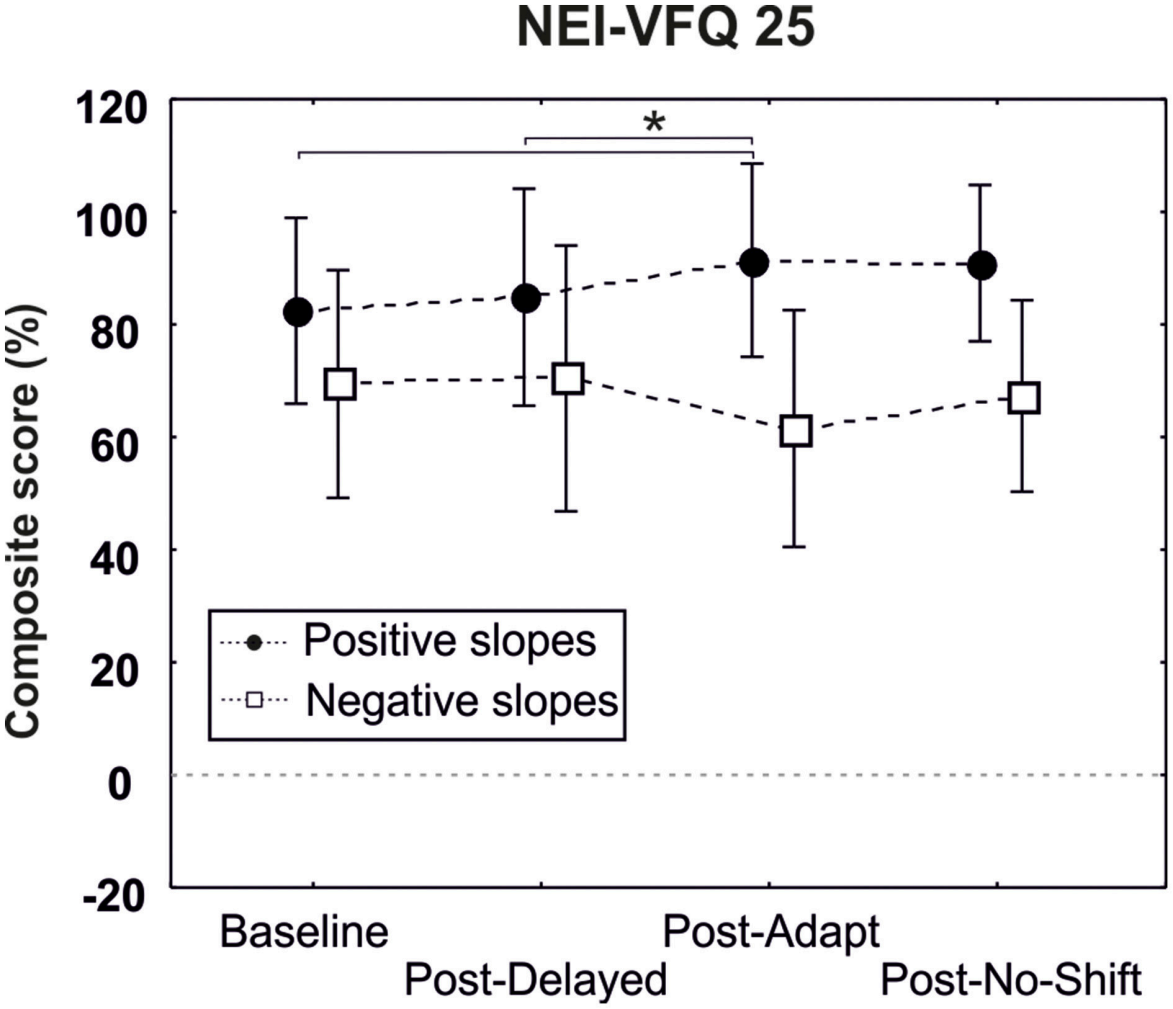
**Results of the questionnaire NEI-VFQ25**. Composite score of patients with a macular spare superior to 5° are represented separately for patients having positive (black circles) and negative (white squares) slopes. Bars represent the confident interval at 0.95. Star represents the significant difference of the Baseline (pre-delayed-shift training) and the post-delayed training as compared to the post-adaptation training, as revealed by *Post-hoc* LSD Fisher test.

One-way repeated measures ANOVA revealed a significant improvement of the composite score following the adaptation training (composite score 91.41%) as compared to the baseline pre-delayed-shift training (composite score 73.48%, *p* = 0.005) and post-delayed-shift training (composite score 84.84%, *p* = 0.03) only for the patient presenting a positive slope and having a macular spare superior to 5° [3 levels interaction Training × Macular spare × Slope; *F*_(3, 18)_ = 4.99, *p* = 0.01] (Figure 7).

## Discussion

In this study, we aimed to test whether a procedure of adaptation of anti-saccades in hemianopic patients could improve their performance in visual exploration, reading and quality of life. To test the specificity of the adaptation component of this procedure, we also designed two control tasks in which subjects simply performed AS without adaptation (Delayed-shift, No-shift). While AS adaptation procedure has been validated in healthy subjects (Lévy-Bencheton et al., 2013), only nine among 14 patients showed the expected increase of AS gain (positive slope). Overall, we found that all three trainings significantly improved visual exploration (decreased RT) in the entire group of patients. In addition, all training improved the reading speed in right-HVFD patients. These functional improvements were not associated to a training-specific increase in saccade amplitude. We, however, found effects specific to the adaptation training, in patients showing a positive slope. First, a significant improvement of reading speed in left-HVFD patients, associated with specific changes of leftward (return) saccades (increased amplitude and decreased number). These effects of the brief adaptation training in left-HVFD patients were maintained 1 month later. Second, an increase of the saccadic amplitude was observed in the Serial exploration task for patients who increased their AS amplitude during the adaptation training. Finally, patients with a macular spare superior to 5° and a positive slope demonstrated a specific improvement of the visual quality of life score following the adaptation training.

In the next paragraphs we will discuss (1) the improvement of functional measures obtained following the AS training; (2) the improvement of oculomotor measures obtained specifically following the AS adaptation training; (3) finally, the usefulness of AS adaptation as a potential rehabilitation method and the different ways to increase its efficiency and long-term after-effects.

### Functional measures: effects of anti-saccade training paradigms?

Our results suggest that the 15 min of anti-saccade training is by itself sufficient to allow right-HVFD patients to read faster and all patients to explore faster. Indeed, after any of the three trainings, we observe a 4% improvement of reaction time in the Pop-out exploration task, a 7% improvement of reaction time in the Serial exploration task, and a 12% improvement of reading speed for the right-HVFD patients (Table 4). Due to absence of consensus about methods to evaluate the functional outcomes in the rehabilitation of HVFD (Bouwmeester et al., 2007), it is difficult to compare these findings to the literature. For example, several studies found a 46–48% improvement of visual exploration reaction time (according to Figure 5 of Zihl, 1995b), but testing only the blind side (Roth et al., 2009) or the best responsive subgroup (Pambakian et al., 2004; Keller and Lefin-Rank, 2010). Other studies revealed an improvement of reading performance ranging from 18 to 58% (Spitzyna et al., 2007) or from 53 to 96 WPM for right-HVFD patients (Zihl, 1995a; Keller and Lefin-Rank, 2010). However, as compared to ours, these studies were based on long training duration, on recruitment starting 3 months after the lesion and / or on outcomes evaluation restricted to the material used during training. Still, our results are encouraging since they reveal significant effects, albeit smaller, for both visual exploration and reading following a very brief (15 min) training session.

**Table 4:** Percentage of modification ((post-phase – pre-phase)/pre-phase)^*^100 (± SD) of functional measures for all tasks, separately for each training, and averaged over all the training (right column).

	**Delayed-shift**	**Adaptation**	**No-shift**	**All training**
**POP-OUT**				
Reaction time	10.48	5.01	–3.59	3.97
	(±12.57)	(±10.51)	(±11.69)	(±12.76)
**SERIAL**				
Reaction time	8.93	10.99	1.92	7.28
	(±7.67)	(±9.69)	(±13.09)	(±10.87)
**READING**				
Word per minute (all)	3.06	17.68	10.17	10.30
	(±21.37)	(±14.12)	(±21.34)	(±19.68)
Word per minute Left-HVFD	–5.30	24.20	4.17	7.69
	(±28.69)	(±12.98)	(±25.42)	(±25.10)
Word per minute Right-HVFD	8.29	13.61	13.92	11.94
	(±15.22)	(±14.01)	(±19.22)	(±15.80)

Positive (negative) values represent an improvement (decline) of performance in post-phase relative to pre-phase.

Improvement of performance after a short-lasting saccadic training has already been observed in a controlled study (Jacquin-Courtois et al., 2013) using a 30 min training in a target ramp-step paradigm eliciting a sequence of pursuit and saccadic ocular movements toward the blind hemifield. Results disclosed a 23% improvement of the performance in an ecological version of Serial exploration task. In both their training paradigm and ours, a visuo-spatial cueing provided by the ramp target presented foveally and the static target presented in the healthy hemifield, respectively, provided the patient with information about the location of the target in the blind hemifield. Such cueing may crucially contribute to boost the performance and shorten the duration of the compensatory training in patients with HVFD. Note however that the cueing cannot explain the long-term and generalized benefit found in the present study, since in Jacquin-Courtois et al. (2013) the improvement shown for Serial-like exploration tasks did not generalize to Pop-out exploration and reading.

Previous studies have demonstrated that the training-related improvements in reading and visual exploration are highly specific and task-dependent (Schuett et al., 2009, 2012; Jacquin-Courtois et al., 2013). However, we demonstrate in the present study an effect of anti-saccade training on both reading (for right HVFD) and visual exploration tasks. Such generalization of saccadic training to different visual tasks has already been shown in a paradigm using an audio-visual training of 4 h daily over a period of 2 weeks in which the patient had to detect a visual stimulus in the blind hemifield, simultaneously presented with a temporally and spatially coincident sound (Passamonti et al., 2009; Keller and Lefin-Rank, 2010). In this audio-visual training, the authors suggested that the generalization was mainly due to low-level neural mechanisms (Bolognini et al., 2005): i.e., by using a multi-sensory integration paradigm their approach reinforces the activation of subcortical structures, specifically the Superior Colliculus, and of cortical areas which contribute to its multisensory activity (Passamonti et al., 2009).

Anti-saccades are not supposed to be based on low-level neural mechanisms but instead involve a large frontal network such as Frontal Eye Field (FEF), Supplemental Eye Field, and Dorsolateral Prefrontal Cortex (Everling and Munoz, 2000; Munoz and Everling, 2004; Pierrot-Deseilligny et al., 2004; McDowell et al., 2008). HVFD being due to either occipital or optic radiations lesions and the neural network involved in eye movements, including cortical areas described above for AS being usually spared (Nelles et al., 2007, 2009), we rather speculate than this extensive network might favor the generalization of AS training to other kind of saccades like those involved in reading and visual exploration in which FEF is also called for (Gitelman et al., 2002; Heinzle et al., 2010). We speculate that thanks to the use of this large network, this might have influenced and boosted high cognitive functions required for perceptual task.

### Are oculomotor measures changes specifically related to as plasticity mechanisms?

Despite a global improvement of functional measures in the visual tasks following all three trainings, the spatial (amplitude) or temporal (fixation) parameters of associated eye movements did not systematically change, in contrast to reports of previous controlled studies (Zihl, 1995a; Spitzyna et al., 2007; Passamonti et al., 2009). Zihl (1995a,b) has shown that repetitive sessions of saccadic training are necessary to improve eye movements in both reading and visual exploration tasks. In our study, the single training session might have been insufficient to trigger significant reorganization of eye movements. Furthermore, the observed dissociation between significant functional performance and absent oculomotor changes suggests that anti-saccade training might stimulate visuo-attentional functions. Changes in oculomotor measures were observed only following the adaptation training, a 15 min training which was sufficient to induce an amplitude increase of saccades in nine patients that could also be observed in the Serial exploration task and, for left-HVFD patients, in the reading task. These fast oculomotor changes suggest the involvement of the specific plasticity mechanisms elicited during saccadic adaptation, as discussed in the following.

First of all, we could not demonstrate a robust effect of the anti-saccade adaptation paradigm in the entire group of patients with HVFDs, as measured by the slopes of AS gain vs. trial number relationship during the training. However, a sub-group of nine patients showed a positive slope of the AS gain in the adaptation training, independently of their side of HVFD and their macular spare. In this sub-group, we could also demonstrate, and specifically following the adaptation training, an increase of saccade amplitude during the reading task (left-HVFD patients) and the Serial exploration task. Note however that patients of this sub-group did not show any change of saccades in the Pop-out task, as well as the right-HVFD patients in the reading task. We did not evaluate the laterality in our patients. However, the only difference between left and right-HVFD patients concerns the reading task, which we believe are related to reading direction rather than to hemispheric asymmetry or laterality. Furthermore, given the voluntary nature of anti-saccades, the stronger transfer of AS adaptation to saccades of the Serial exploration task than to saccades of the Pop-out exploration task is consistent with the fact that saccades in the Serial exploration task are triggered on a more voluntary basis than in the Pop-out exploration task.

In the reading task, right-HVFD patients did not present any oculomotor changes while left-HVFD patients did. Several arguments from the saccadic adaptation literature can explain this difference. First, according to the notion of adaptation field (Frens and van Opstal, 1994), adapting one single saccade transfers more to saccades of larger amplitude than to saccades with a shorter amplitude (Schnier et al., 2010). Since we adapted AS of 6° to 12°, this could explain the lack of transfer to rightward reading saccades of around 3–4° in amplitude for right-HVFD patients and the presence of transfer to leftward return saccades (around 25°) for left-HVFD patients. Another explanation is that the lack of after-effect on rightward saccades during the post-phase reading task results from a faster de-adaptation, due to the large number of saccades performed toward this direction as compared to leftward saccades (Alahyane and Pelisson, 2005). Finally, a last explanation would be that right-HVFD patients would need to train twice more than the left-HVFD to show oculomotor changes, as Zihl suggested in a previous study (Zihl, 1995a). We should however keep in mind that the effects are demonstrated on a small group of patients (left-HVFD patients), and that such effects should be further tested and reproduced in a larger group of patients.

Finally, the neural underpinnings of the functional benefits related to the involvement of specific plasticity mechanisms elicited during the AS adaptation training has still to be determined. Indeed, beyond the classical contribution of the cerebellum, the neural substrates of saccadic adaptation remains largely unknown, notably those of AS adaptation. Recent studies using fMRI (Blurton et al., 2012; Gerardin et al., 2012) or TMS (Panouilleres et al., 2014) in healthy subjects have revealed the involvement of parieto-frontal areas of the cerebral cortex in adaptation of reactive saccades and of scanning saccades, and studies in patients with a thalamic lesion provided evidence for a role of the cerebello-thalamo-cortical pathways for reactive saccades adaptation (Gaymard et al., 2001; Zimmermann et al., 2015). Concerning anti-saccades, our study demonstrating the possibility to induce AS adaptation in healthy subjects (Lévy-Bencheton et al., 2013) is to our knowledge the only study on AS plasticity so far. In this study, we speculated that the frontal cortex and its recurrent connections with the basal ganglia could be the locus of AS adaptation. According to this hypothesis, AS adaptation training in the present study could have changed the activity of these basal ganglia-frontal systems in HLH patients and led to functional improvements in tasks (reading, serial visual exploration) requiring not only accurate oculomotor control but also efficient “frontal functions” such as cognitive flexibility and short-term working memory.

### Anti-saccade adaptation as a potential rehabilitation method

The patients who showed an increase of the anti-saccade gain during the adaptation training (i.e., positive slope) also demonstrated some specific functional and oculomotor effects in the reading task. That specificity for the same subgroup of patients (i.e., positive slope) is also observed in the composite score of the quality of life questionnaire, which is significantly increased following the adaptation training for patients presenting a macular spare superior to 5°. This suggests that using outward adaptation of anti-saccades is a promising paradigm, whose effect might be further tested in more repetitive training.

The first advantage of such paradigm compared to previous ones is that saccadic adaptation is induced fast and effortless. The first study aiming at testing such an automatic strategy on reading performance showed that following a 15 h training (in right-HVFD only) patients presented a 18% increase of their reading speed (Spitzyna et al., 2007). In our study, for the sub-group of patients with a positive slope (i.e., “adapted-group”) a significant improvement up to 24.20% was found in left-HVFD patients. This result is quite remarkable given the use of a single and short (only 15 min) session of eye movement training. For this reason, we think that adding an automatic component (adaptation) to the anti-saccade training improves the possibility to enhance performance.

Additionally, patients presenting a positive slope during the adaptation training seem to feel the benefice 1 month after the training as demonstrated by the specific increase of their composite score in this condition. Most importantly, the results reported that this efficiency occurs only for patients presenting a macular spare superior to 5° suggesting that they might have better facilities to detect the feedback target, (as compared to patients presenting a macular spare inferior to 5°), thus reinforcing the effect of plasticity mechanisms. However, it is important to underline that patients presenting a macular spare inferior to 5° do adapt as well, as suggested by their inclusion/presence in the subgroup of nine patients. Despite the small results, albeit promising, demonstrated in the post-phases during the computerized tasks (i.e., reading and visual explorations tasks) we should keep in mind that the patients explicitly reported an enhancement in their daily life activities and, this measure, although subjective, should be all the more taken into account in all studies focusing on rehabilitation methods.

Finally, even though our saccadic adaptation paradigm failed to induce functional effects on reading for right-HVFD patients, and in visual exploration tasks (although oculomotor changes occurred in the Serial exploration task), we suggest that a longer adaptation training would allow right-HVFD patients to reach the same level of performance than left-HVFD patients (Zihl, 2000). Repeating the 15 min protocol over 2 weeks could also help to increase the generalization of transfer to different visual tasks (Bolognini et al., 2005; Passamonti et al., 2009; Keller and Lefin-Rank, 2010) and its long term retention (Passamonti et al., 2009; Wang et al., 2012).

## Conclusion

We demonstrate for the first time that 15 min of an anti-saccade adaptation training improves both reading and visual exploration tasks. Furthermore, all patients having a macular spare superior to 5° still benefit from the training at 1 month, as evaluated by a questionnaire on quality of life. Taken together, we believe that AS adaptation training, with some suggested improvements, could become an efficient and costless rehabilitation tool for patients suffering of HVFD.

## Author contributions

DL, LP, DP, CT, and SJ contributed to the design of the experiments. RS designed saccadic program aiming at presenting computerized tasks on the screen. DL and MP performed experiments and conducted data analysis. DL, LP, DP, and CT interpreted the data. DL, LP, DP, CT, and SJ drafted, wrote, and approved the final version of the manuscript. CT supervised the project.

### Conflict of interest statement

The authors declare that the research was conducted in the absence of any commercial or financial relationships that could be construed as a potential conflict of interest.
